# Full tensor registration of diffusion tensor magnetic resonance imaging for assessment of cardiac pathologies

**DOI:** 10.1186/1532-429X-14-S1-W41

**Published:** 2012-02-01

**Authors:** Carla S Gil, Stephen D Meredith, Kathleen M Curran

**Affiliations:** 1 School of Medicine and Medical Science, University College Dublin, Dublin, Ireland

## Background

Cardiac fiber architecture can be characterized through DTI, resulting in a better understanding of the electrical and mechanical behavior of the heart. Structural remodeling of the myocardium may also be observed. Most cardiac DTI studies are performed ex-vivo and only recently have in-vivo measurements become possible. However, in-vivo imaging remains challenging due to signal attenuation from heart motion. Previous research to create a cardiac DTI atlas used registration of scalar images such as fractional anisotropy maps and anatomical MRIs. This study aims to register the DT images using an algorithm that relies on the full tensor information and tensor orientation to drive the registration in order to compare a population of normal hearts with a population of hearts with dyssynchronous heart failure.

## Methods

Data Acquisition: 7 normal and 4 failing canine hearts were imaged using DTI with the long axis aligned with the scanner’s z-axis. Diffusion was measured on a 1.5T GE scanner in a minimum of 16 different directions, b-value = 1500s/mm2 and FOV varied from 8 to 10cm. Data was provided by the Center for Cardiovascular Bioinformatics and Modeling.

DTI Registration: Our registration algorithm matches the tensor orientation to find the registration transformation. The source was warped into alignment with the target by applying a transformation that matches tensor size, shape and orientation most closely. Images were aligned using affine transformations and the Preservation of Principal Direction (PPD) reorientation strategy. Registration was performed with global optimization using Powell’s local optimization combined with Simulated Annealing global optimization.

## Results

Our previous work concluded that similarity measures sensitive to the full tensor perform better than scalar derived measures when registering inter-subject DTI. The 7 normal hearts were registered to a template (one of the normal hearts) using the principal direction as a similarity measure. The failing hearts were then registered to the mean normal DTI. The inclination angle, defined as the angle between the principal eigenvector and the short-axis plane, was also computed.

## Conclusions

DT image registration using the full tensor information has been shown in the literature to provide more accurate results for the human brain. Our preliminary study confirms these findings for Cardiac DTI registration. By creating an atlas with a better image alignment, more accurate comparisons with DT images of cardiac pathologies could be obtained which would be useful for clinical assessment of cardiac pathologies.

## Funding

This work is funded by a Research Frontiers Grant (09/RFP/CMS2408) awarded by the Science Foundation Ireland.

We would like to acknowledge Drs. Patrick A. Helm and Raimond L. Winslow at the Center for Cardiovascular Bioinformatics and Modeling and Dr. Elliot McVeigh at the National Institute of Health for provision of data.

